# Hybrid Genome Assembly and Annotation of the Basidiomycete Fungus *Candolleomyces candolleanus* Strain CMU-8613 Using a Cost-Effective Iterative Pipeline

**DOI:** 10.3390/ijms27010509

**Published:** 2026-01-03

**Authors:** Edgar Manuel Villa-Villa, Ma. Soledad Vázquez-Garcidueñas, Gerardo Vázquez-Marrufo

**Affiliations:** 1 Centro Multidisciplinario de Estudios en Biotecnología, Facultad de Medicina Veterinaria y Zootecnia, Universidad Michoacana de San Nicolás de Hidalgo, Km 9.5 Carretera Morelia-Zinapécuaro, Col. La Palma, Tarímbaro CP 58893, Mexico; edgar.manuel.villa@umich.mx; 2 División de Estudios de Posgrado, Facultad de Ciencias Médicas y Biológicas “Dr. Ignacio Chávez”, Universidad Michoacana de San Nicolás de Hidalgo, Ave. Rafael Carrillo esq. Dr. Salvador González Herrejón, Col. Cuauhtémoc, Morelia CP 58020, Mexico; soledad.vazquez@umich.mx

**Keywords:** *Candolleomyces*, genome, hybrid assembly, pipeline performance comparison, BGCs, CAZymes, mating type

## Abstract

The recently described genus *Candolleomyces* (Basidiomycota, Agaricales, Psathyrellaceae) is now recognized as a distinct taxonomic group separate from *Psathyrella*. Currently, no fully assembled and accurately annotated genomes of *Candolleomyces* species are available, limiting our understanding of their physiological traits and biotechnological potential. Numerous tools exist for fungal genome assembly and annotation, each using different algorithms, resulting in substantial variation in gene content and distribution within the same genome. In this work, a hybrid assembly and annotation of the genome of strain CMU-8613 were performed using pipelines that combine different assembly and annotation tools. Phylogenetic analysis showed that the analyzed strain CMU-8613 belongs to *Candolleomyces candolleanus*. The assembled genome size ranged from 46.8 Mb (NECAT + Racon) to 59.3 Mb (Canu + *Coprinellus micaceus* genome assembly), depending on the assembly and polishing strategy. The analysis identified 15–25 secondary metabolite gene clusters (BGCs), depending on the genome assembly and the tools used for BGC prediction. In strain CMU-8613, CAZyme-encoding genes varied across assemblies: 494 genes were detected in the Flye assembly and 453 in NECAT; in both cases, the AA (Auxiliary Activities) and GH (Glycoside Hydrolases) families were the most represented. The diversity of CAZymes observed among *Candolleomyces* species suggests differences in their saprophytic capacities. Analysis of the MAT-A/MAT-B loci revealed that *C. candolleanus* possesses a tetrapolar mating system. This study provides the first annotated genome of *C. candolleanus*, highlighting its enzymatic potential to degrade plant biomass and its capacity to synthesize diverse secondary metabolites. The combination of assembly and annotation tools employed here offers robust alternative strategies for characterizing non-model fungi or species lacking high-quality reference genomes.

## 1. Introduction

The genus *Candolleomyces* was recently established by separating it from the polyphyletic genus *Psathyrella*, based on a robust multilocus phylogenetic analysis and the absence of the characteristic pleurocystidia of *Psathyrella sensu stricto* [1,2]. Currently, the genus comprises 63 species, of which 68.33% are reported from Asia, 18.33% from Europe, 10% from North America, and only 1.66% from Africa and South America [2]. Species of this genus are saprotrophic and contribute to the decomposition of dead plant material in temperate and tropical forests [3]. This ecological role suggests high biotechnological potential for the degradation of lignin, cellulose, and hemicellulose [4]. Additionally, several antioxidant and antibacterial compounds have been characterized from strains of *Psathyrella candolleana*, now considered *Candolleomyces candolleanus* [5]. Chloroform extracts of the basidiocarp inhibit Gram-positive bacteria *Staphylococcus epidermidis* and *Streptococcus pneumoniae*, both associated with respiratory infections [6]. Furthermore, the ethyl acetate extract obtained from mycelium and the extracellular filtrate from Czapek–Dox liquid culture medium exhibit antibacterial activity against *Staphylococcus aureus* and antioxidant properties [7].

To date, most characterized secondary metabolites in *P. candolleana* (hereafter considered a synonym of *C. candolleanus*) are diterpenes. Among these, psathyrelloic acid I is a monocyclic compound with antibacterial activity against *S. aureus* [8]. Subsequently, two tetracyclic diterpenoids, psathyrins A and B, were identified and showed antibacterial activity against *S. aureus* and *Salmonella enterica* but not against *Pseudomonas aeruginosa* [9]. More recently, five guanacastane-type diterpenes (psathyrellins A–E) were isolated; psathyrellins A, B, and C showed antibacterial activity against *Escherichia coli*, *S. aureus*, *S. enterica*, and *P. aeruginosa* [10]. Importantly, none of these studies includes molecular identification or phylogenetic analysis of the strains used to isolate the compounds, a relevant omission given the recent taxonomic reclassification of the previously mentioned genus.

Despite their ecological relevance and biotechnological potential, genomic information for *Candolleomyces* species remains scarce. This lack of genomic data extends to other taxa within the Psathyrellaceae family, for which only 17 genomes are currently available in the National Center for Biotechnology Information (NCBI) and the Joint Genome Institute’s MycoCosm database (accessed 11 May 2025), and only three correspond to *Candolleomyces* species. Recently, the genome of a strain identified as *P. candolleana* was analyzed, revealing a gene cluster associated with guanacastane diterpene biosynthesis; however, this genome has not been deposited in public repositories [11]. The limited number of high-quality assembled genomes for *Candolleomyces* restricts comparative genomic analyses aimed at identifying genes of biotechnological interest and exploring evolutionary patterns associated with its saprotrophic lifestyle.

Here, we report the assembly and annotation of the genome of strain CMU-8613, isolated in central Mexico, using a hybrid strategy that combines short Illumina reads with long Oxford Nanopore reads. We also provide alternative assembly and polishing pipelines for cases where no reference genome is available. Finally, we discuss the biological and biotechnological implications of the identified genomic features.

## 2. Results

### 2.1. Primary Assembly

The primary assembly is a crucial step in reconstructing the genome of a fungal species. In this study, the tools Canu, Flye, and NECAT were used, which are among the most widely used for fungal genome assembly and are designed to perform initial assembly from long ONT reads. Each tool relies on different algorithmic approaches, producing assemblies with distinct characteristics. To evaluate the primary assemblies generated by these three tools, we compared standard metrics from BUSCO analyses, including gene completeness, N50, contig numbers (a measure of contiguity), and total genome size. Canu and NECAT require an estimated genome size as input. In contrast, Flye no longer needs this information starting from version 2.6, making it well-suited for species lacking a reference genome. Because no reference genome was available for the strain analyzed in this study, the approximate genome size obtained from the initial Flye assembly (~60 Mb) was used as the input size for Canu and NECAT.

The comparative analysis revealed significant differences in the continuity and completeness of the primary assemblies generated by the three strategies. The Flye assembly achieved the highest percentage of complete BUSCOs, indicating better recovery of conserved gene content. Canu produced a similar level of completeness but with a slightly higher duplication rate, suggesting challenges in resolving repetitive or heterozygous regions. Additionally, Canu produced the least contiguous assembly, with an N50 of 95 kb and many contigs, indicating considerable fragmentation. In contrast, NECAT produced the most contiguous assembly, with the highest N50 and the fewest contigs. However, NECAT also exhibited the lowest gene completeness, the highest number of missing BUSCOs, and the smallest total genome size. This reduction in size and completeness likely results from more aggressive collapsing of repetitive regions or sequence loss during error correction and assembly. Overall, the Flye assembly was intermediate in both contiguity and completeness (Table 1).

The results from the primary assemblies highlight a common trade-off in fungal genome reconstruction: maximizing gene recovery (completeness) often comes at the expense of structural contiguity, and vice versa. If completeness is the primary goal, Flye and Canu are the best tools; however, if contiguity is more critical, NECAT is the better choice. Because both completeness and contiguity are crucial for subsequent polishing and annotation, all three assemblies were included in the downstream steps to improve contiguity without compromising gene completeness.

### 2.2. Genome Polishing

**Figure 1:**
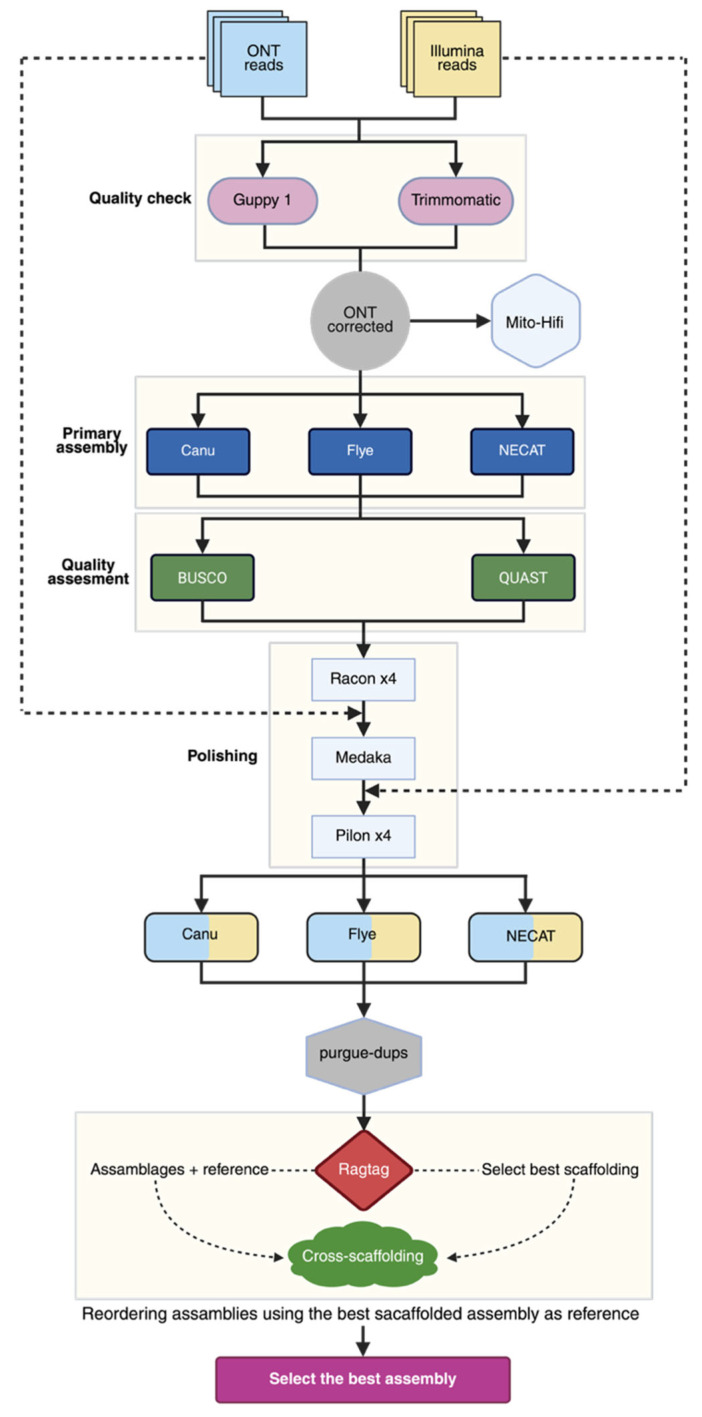
Strategy used for the assembly and polishing of the genome of strain CMU-8613. For details, see the text. Created in BioRender. Vázquez-Marrufo, G. https://BioRender.com/phr8jsz (accesed 24 November 2025).

Genome polishing is an essential step for correcting errors in assemblies generated from long-read data, especially those from ONT sequencing, which often contain residual nucleotide-level errors. In this study, we used a strategy that combined individual polishing tools and sequential tool combinations. Four polishing pipelines were applied to the main assemblies, as described below and shown in Figure 1.

1. Racon: Uses the original long reads to build a consensus based on partial-order graphs (POA) and correct errors;
2. Pilon: Uses aligned Illumina short reads for the assembly to correct specific errors (SNPs and small indels) with high accuracy;
3. Racon + Medaka (R+M): Combines Racon with Medaka, another long-read polishing tool that uses neural networks and is optimized for ONT data, aiming to improve consensus accuracy;
4. Racon + Medaka + Pilon (R+M+P): A hybrid approach where Racon and Medaka are first used to improve the long-read consensus, followed by Pilon with short reads for fine base-level correction.

Racon improved assembly continuity by increasing the N50 and reducing the number of contigs relative to the unpolished assemblies. However, it did not substantially enhance base-level accuracy, as reflected in the BUSCO scores, which were not among the highest and indicate a residual error rate for this method (Table 2). Pilon, in turn, produced a slight improvement in BUSCO completeness for the Flye assembly but had a markedly adverse effect on contiguity, significantly increasing the number of contigs and reducing the N50 value.

The R+M (Racon + Medaka) combination maintained excellent contiguity while improving BUSCO completeness by reducing the number of missing genes compared with Racon alone. The most balanced approach was the R+M+P strategy, which yielded the highest percentages of complete BUSCOs and the fewest missing BUSCOs—particularly for the NECAT assembly. This combined pipeline also maintained high contiguity, benefiting from Pilon’s base-level corrections without the fragmentation observed when Pilon was applied individually. Although the R+M+P pipeline performed optimally for both initial assemblies, the results differed between them. The Flye assembly consistently achieved higher completeness than NECAT, even after optimal polishing. This indicates that the quality and content of the primary assembly set an upper limit that polishing can refine but not surpass. In other words, decisions made during the primary assembly stage propagate through the entire workflow and fundamentally shape the outcome.

### 2.3. Framework Using the Genome of Coprinellus Micaceus as an External Reference

**Figure 2:**
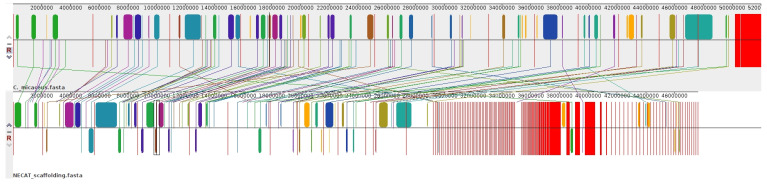
Genome-wide synteny between *Coprinellus micaceus* and the CMU-8613 strain of *Candolleomyces candolleanus*. The whole-genome alignment, generated with Mauve, shows the reference genome (*C. micaceus*) on the upper track and the scaffolded NECAT assembly of CMU-8613 on the lower track. Colored blocks represent Locally Collinear Blocks (LCBs), highlighting regions of conserved sequence homology without rearrangements. Connecting lines indicate orthologous regions, demonstrating a high degree of macrosynteny between the two species.

Given the lack of a high-quality reference genome for *Candolleomyces* or closely related species, we used the available genome of *Coprinellus micaceus* (GenBank: GCA_951394405.1) as an external reference. This species belongs to the family Psathyrellaceae and is the closest phylogenetic genus with an annotated genome. Although not ideal, this approach can still provide sufficient information to order and orient a portion of the contigs. To validate *Coprinellus micaceus* as a structural reference, we performed a whole-genome synteny analysis with the CMU-8613 strain of *C. candolleanus* using Mauve. The alignment revealed extensive conservation of genomic architecture, with large Locally Collinear Blocks (LCBs) shared between the genomes. As shown in Figure 2, scaffolding effectively resolved contig orientation, demonstrating a high degree of macrosynteny. Despite the evolutionary divergence between the two genera, the conserved chromosomal organization supports using *C. micaceus* as a guide for contig ordering in the CMU-8613 genome.

The scaffolding process improved the continuity of all three assemblies by increasing scaffold N50 values relative to contig N50 values and by reducing the total number of scaffolds (Table 3). This improvement can be attributed to the considerable macrosynteny between *C. micaceus* and CMU-8613 genomes. The NECAT assembly showed the greatest improvement, achieving a scaffold N50 of 1 Mb—substantially higher than that of the Flye and Canu assemblies. This suggests that NECAT’s inherently higher contiguity facilitated ordering relative to the reference. Importantly, BUSCO metrics remained nearly unchanged after scaffolding. The slight increase in total assembly size likely reflects the introduction of ‘N’ characters to represent gaps between contigs within scaffolds. The stability of BUSCO scores confirms that scaffolding reorganized and oriented the existing contigs without substantially altering or recovering core gene content. Despite the enhanced continuity, some limitations remain. NECAT produced the most contiguous assemblies, but also the least complete and smallest. Canu remained the most fragmented at both the contig and scaffold levels, yielding the highest number of scaffolds. Flye retained its high completeness while achieving intermediate continuity.

### 2.4. Iterative Scaffolding Using the NECAT Assembly as a Reference

**Figure 3:**
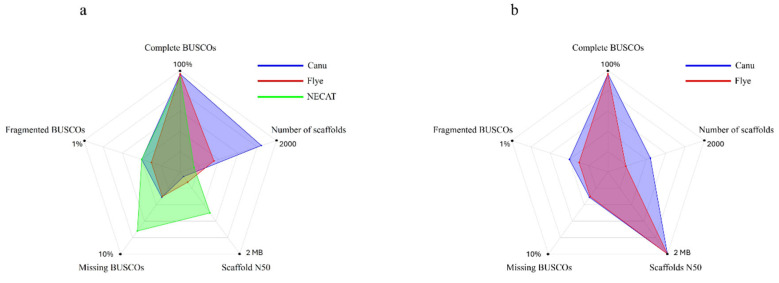
Representation of the BUSCO scores in the genome assemblies of the CMU-8613 strain, obtained using: (**a**) the genome of *Coprinellus micaceus* (GCA_951394405.1) as a reference and (**b**) NECAT.

Given the high continuity of NECAT, the greater completeness of Flye and Canu, and the limitations of the external reference—since it is not from the same species or genus—a strategy of iterative scaffolding was implemented. The NECAT assembly, improved in continuity through scaffolding with the *C. micaceus* genome (Scaffold N50 > 1 Mb), served as a reference to order and orient the primary assemblies of Canu and Flye. The goal of this strategy was to combine the strengths of different assembly approaches to transfer the continuity achieved in the NECAT assembly to the Canu and Flye assemblies, which were more complete but more fragmented. The results are presented in Table 4. The difference between the two orderings is shown in Figure 3.

This strategy was effective, as the initial assemblies with Canu and Flye showed a significant increase in contiguity, reaching an N50 of 2 MB for scaffolds, an improvement over the N50 obtained using the external reference *C. micaceus* (Table 4). The number of scaffolds also decreased drastically, especially in Flye (from 771 contigs to 370) scaffolds.

Again, the BUSCO scores remained stable, indicating that the improvement was structural (Table 4, Figure 3). This result demonstrates the potential of using an internally generated, contiguity-optimized assembly as a scaffold for other assemblies in the same project, prioritizing completeness. This approach can be beneficial for non-model organisms, where high-quality external references are lacking, because it allows leveraging the complementary strengths of different assembly algorithms iteratively.

Among the two assemblies produced by this second scaffolding step, the one generated by Flye proved to be the most promising. It maintained its initial completeness (BUSCO: 96.7%) and now shows excellent continuity (Scaffold N50: 2 MB), with a significantly lower number of scaffolds (370) than the assembly derived from assembly (882 scaffolds), despite sharing the same Scaffold N50 value. Therefore, the Flye and NECAT assemblies were selected, and their quality results are shown in Table 5 for the annotation stage.

### 2.5. Comparison of Genome Annotation and Functional Analysis

To evaluate the biological impact of the observed differences in the quality of the final assemblies, both genomes were annotated with the Funannotate gene prediction tool, and the results were compared at structural and functional levels. The annotation results reflect differences in the underlying assembly quality (Table 6). The larger, more complete gene assembly, derived from Flye, enabled the prediction of a significantly greater number of genes, suggesting greater integrity of the gene models, likely due to less fragmentation in coding regions or fewer residual frameshift errors in the assembly. Exon, gene, and protein lengths are very similar, indicating that the differences are more related to the number of predicted features rather than individual sizes (Table 6). This strong correlation between assembly quality metrics, particularly BUSCO and size, and the comprehensiveness of the resulting gene annotation reinforces the value of BUSCO as an early predictive indicator of an assembly’s potential for subsequent biological analyses.

**Figure 4:**
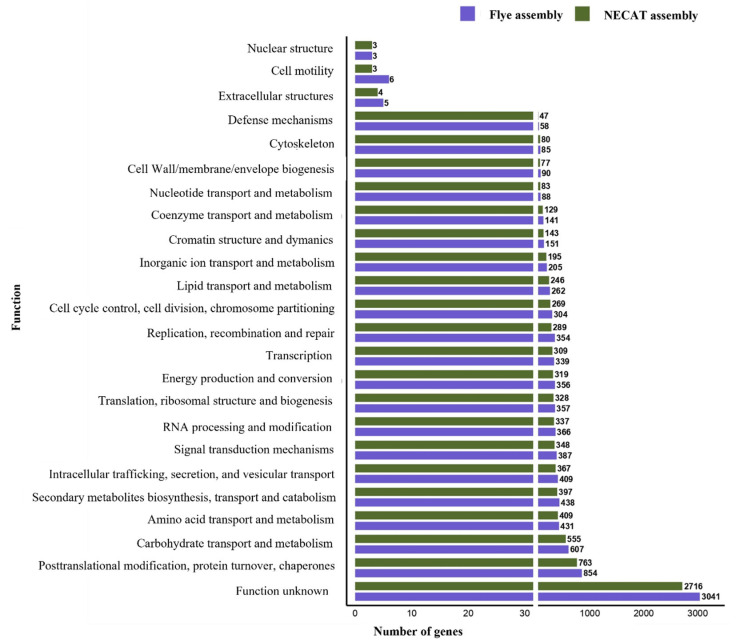
Number of genes annotated by COG functional categories in the genome of strain CMU-8613, *C. candolleanus*. The graph shows the number of genes in the genomes assembled with Flye and NECAT, sorted and polished.

Functional annotation was performed by assigning predicted genes to COG categories and by searching for clusters of secondary metabolite biosynthesis genes (BGCs) using the antiSMASH tool. The COG annotation was consistent with the largest number of predicted genes; the Flye assembly assigned more genes to nearly all COG functional categories than NECAT (Figure 4).

### 2.6. Features of the Mitochondrial Genome

**Figure 5:**
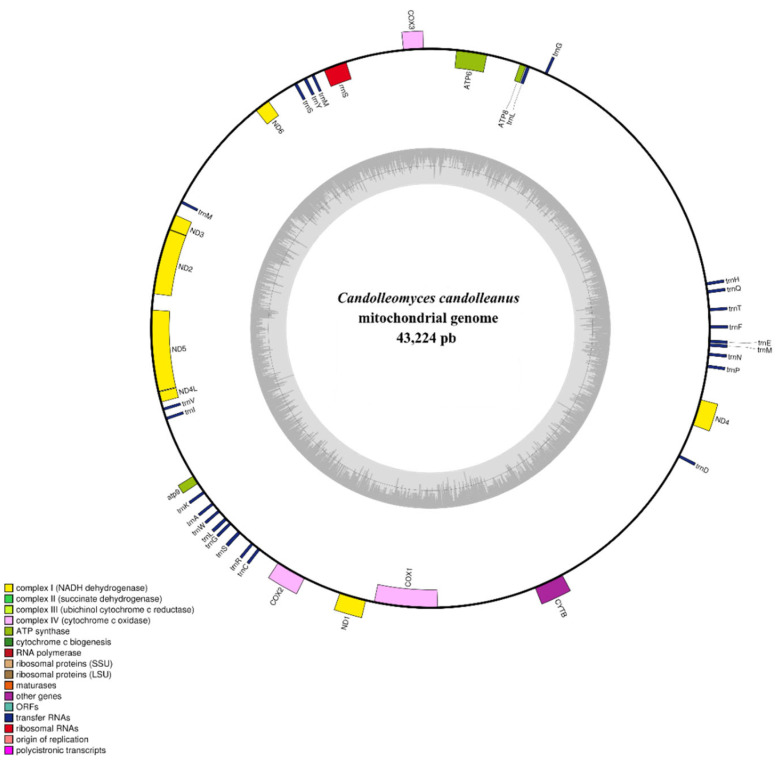
Circular map of the mitochondrial genome of *Candolleomyces candolleanus*. Blocks of different colors represent genes; those outside the outer ring are on the forward strand, while those inside the outer ring are on the reverse strand. The gray-scale bar chart on the inner ring shows the GC content of the mitochondrial sequences. The inner circle of the GC content chart marks the 50% threshold. The complete mitochondrial genome map was created using OGDraw v1.2 [12].

The assembled mitochondrial genome of *Candolleomyces candolleanus* was approximately 43,224 bp in length (Figure 5). Functional annotation using MitoHiFi revealed that it comprises 40 genes, including 13 protein-coding genes (CDS), two ribosomal genes (rnl and rns), and 25 transfer RNA (tRNA) genes.

The coding genes correspond to essential components of the mitochondrial respiratory machinery, including subunits of NADH dehydrogenase complexes (*nad1–nad6*, *nad4L*), cytochrome c oxidase (*cox1*–*cox3*), cytochrome b (*cob*), and ATP synthase (*atp6*, *atp8*, *atp9*). The identified tRNAs cover most amino acids, indicating functional and autonomous mitochondrial translation machinery.

### 2.7. Secondary Metabolite Gene Clusters (BGCs)

**Figure 6:**
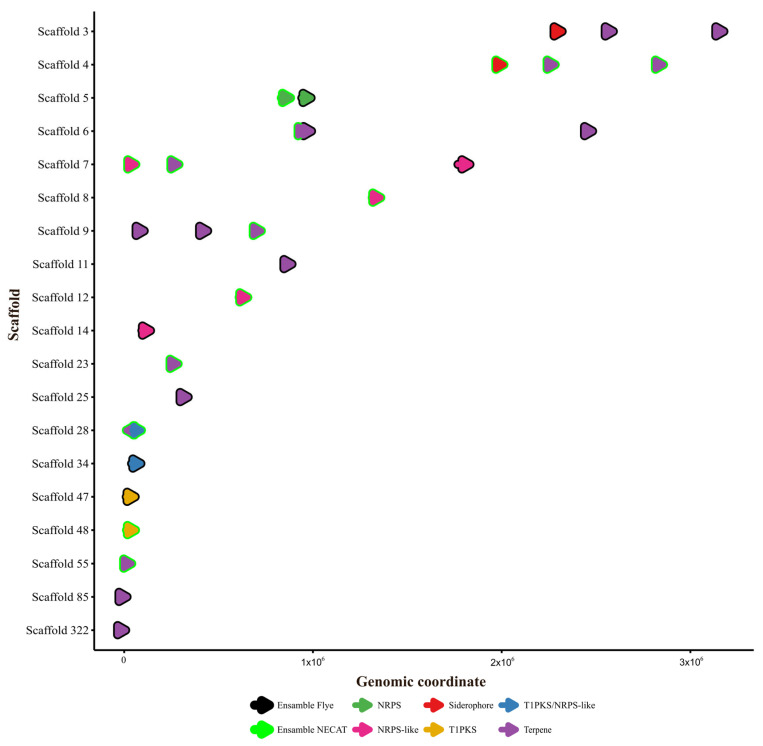
Coordinate map of the BGCs characterized in the polished and ordered assemblies of Flye and NECAT. The overlapping colors of the BGCs indicate that both assemblers place them on the same scaffold.

The antiSMASH tool identified a similar number of BGCs in both assemblies: 16 in the Flye assembly and 15 in the NECAT assembly (Table 7). Although the totals were comparable, the BGCs were distributed across different scaffolds and varied in size (number of bases), as shown in the genetic coordinate map in Figure 6. The types of BGCs identified included terpenoid synthases (10 in Flye, 9 in NECAT), non-ribosomal peptide synthetases (NRPS, 3 in both), polyketide synthases (PKS, 1 in both), siderophore synthesis (1 in both), and a hybrid NRPS-T1PKS cluster in both (Table 8). Similarly, the FunBGCex analysis revealed moderate differences in the number and composition of BGCs detected between the assemblies generated with Flye and NECAT.

In total, the Flye assembly identified 25 BGCs, while NECAT identified 23, suggesting greater recovery of biosynthetic loci in the Flye assembly. However, both assemblies shared a similar biosynthetic profile, including terpene-type clusters (TC), NRPS, PPPS, DMATS, and UbiA, indicating that both methods consistently reconstructed the overall metabolic capacity of the genome.

A comparison of FunBGCex and antiSMASH shows strong agreement on the types of clusters detected, though with differences in sensitivity and boundary definitions. Overall, FunBGCex was more sensitive to partial BGC fragments, while antiSMASH provided a more precise definition of each cluster’s boundaries (Table 7). The assembly generated with Flye stood out in both tools, showing a greater number of BGCs and higher similarity to reference clusters, suggesting that it offers a more complete reconstruction of biosynthetic regions than NECAT (Table 8).

### 2.8. Number of CAZyme-Encoding Genes

**Figure 7:**
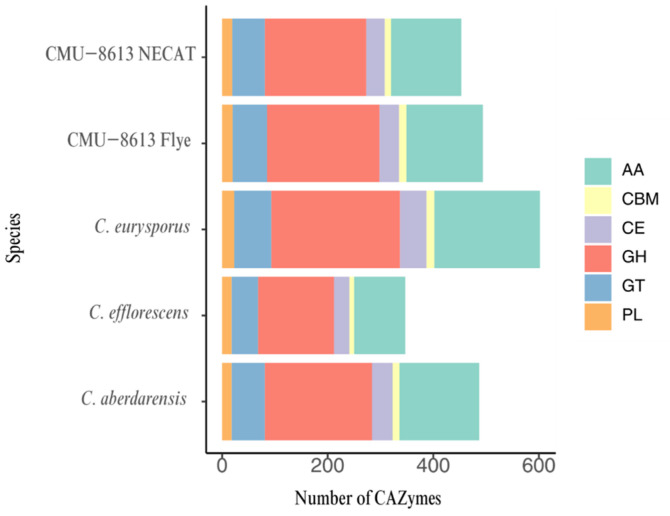
Distribution of CAZyme family genes in *Candolleomyces* species.

Functional annotation analysis using dbCAN identified multiple CAZyme families in the genomes of the four studied *Candolleomyces* species (Figure 7). The detected families spanned the main functional groups of the CAZyme classification, including AA (Auxiliary Activities), CBM (Carbohydrate-Binding Modules), GH (Glycoside Hydrolases), GT (Glycosyltransferases), CE (Carbohydrate Esterases), and PL (Polysaccharide Lyases). The AA and GH families were consistently the most abundant across all four species.

After applying high-confidence filtering, the total number of CAZymes per species varied across the analyzed genomes, with 494 in the Flye assembly and 453 in the NECAT assembly for strain CMU-8613. These results are similar to those of *Candolleomyces aberdarensis*, which had 487, whereas the assemblies of *Candolleomyces efflorescens* and *Candolleomyces eurysporus* showed the most significant differences, with 347 and 602, respectively. This variation in CAZyme gene counts suggests potential functional differences in carbohydrate degradation, modification, or synthesis within the *Candolleomyces* genus. The species *C. eurysporus* had the highest number of genes associated with GH and AA, while *C. efflorescens* had fewer genes related to both enzymatic functions. The numbers of CBM and PL enzymes remain relatively conserved across the compared species (Figure 7). In the specific case of strain CMU-8613, the main difference between the Flye and NECAT assemblies was the location of the GH and AA genes: with the first, 213 and 145 were detected, respectively, whereas with the second, 192 and 133 were detected.

### 2.9. Type of Mating in the CMU-8613 Strain

**Figure 8:**
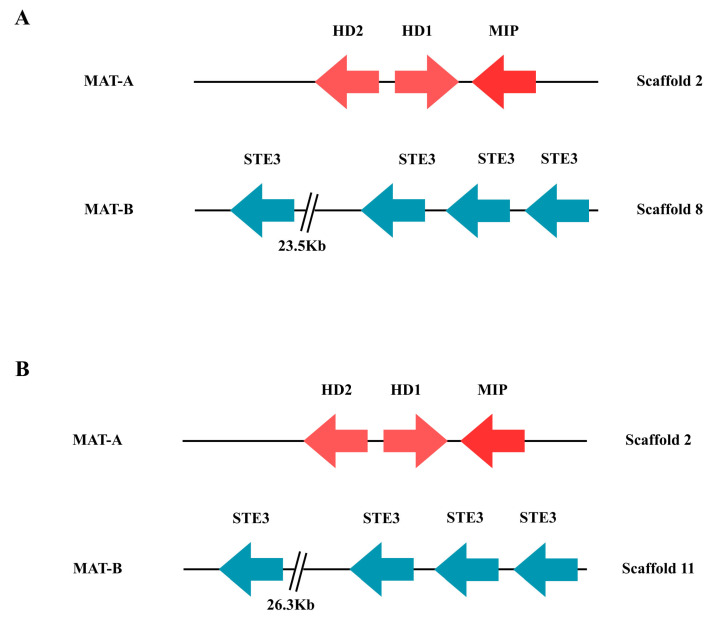
Distribution of mating genes in the CMU-8613 strain of *Candolleomyces candolleanus*. Homeodomain protein HD1, homeodomain protein HD2, MIP mitochondrial interpeptidase, and STE pheromone receptor similar to STE3. (**A**) Flye assembly. (**B**) NECAT assembly. The numbers below each gene correspond to the locus tag in GenBank files.

Functional analysis identified the HD1, HD2, and MIP genes associated with the *MAT-A* locus in *C. candolleanus*, all located on scaffold 2 in both the Flye and NECAT assemblies (Figure 8). This suggests that both methods correctly assemble the homeodomain-encoding region. In contrast, the genes of the *MAT*-B locus, corresponding to GPCR-type pheromone receptors, were found in different genomic regions in each assembly: on scaffold 8 in the Flye assembly and on scaffold 11 in the NECAT assembly (Figure 8).

No additional homeodomain-associated genes were identified outside scaffold 2, confirming that *MAT-A* and *MAT-B* are not physically linked in the analyzed genome. The separation of both loci onto different scaffolds, together with the complete presence of the HD and MIP genes in *MAT-A* and the pheromone receptors in *MAT-B*, is consistent with a tetrapolar reproductive system.

### 2.10. Phylogenetic Analysis

**Figure 9:**
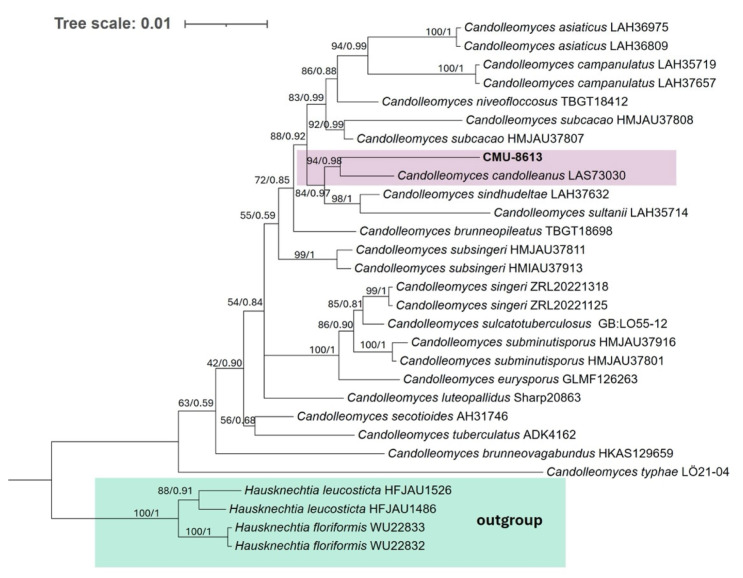
Phylogenetic tree of strain CMU-8613. The clustering pattern was generated using Bayesian inference and the Maximum Likelihood criterion, based on concatenated ITS and LSU sequences. In the ML analysis, TIM2e+G4 (ITS) and TPM3+G4 (LSU) were used. Confidence values from 1000 bootstrap iterations for the ML tree, as well as posterior probability values for the Bayesian phylogeny, are shown at each node (UB/PP). All included species belong to the genus *Candolleomyces* or its synonym *Psathyrella*. Two geographic isolates of two species from the genus *Hausknechtia* were used as an outgroup.

Once the genome assembly was obtained, the ITS region and the LSU gene were used to reconstruct the phylogeny of strain CMU-8613. All species with both sequences available in GenBank were included in the analysis to produce a robust phylogeny. The strain under study clustered with high bootstrap support alongside the type strain Psathyrella candolleana, currently considered *C. candolleanus* (Figure 9).

## 3. Discussion

Species of the genus *Candolleomyces* possess considerable biotechnological potential due to their production of bioactive secondary metabolites of pharmacological relevance. Basic studies aimed at identifying pharmacological activities show that crude extracts of the vegetative mycelium of *Candolleomyces candolleanus* (syn. *Psathyrella candolleana*) exhibit notable antimicrobial and antioxidant properties. This has generated interest in isolating and structurally characterizing the secondary metabolites of *C. candolleanus*, particularly diterpenoids, which constitute the distinctive chemical signature of this species’ secondary metabolism. However, no annotated genome is currently available in public databases to enable a comprehensive assessment of the biotechnological potential of species within the genus *Candolleomyces*.

The phylogenetic analysis places strain CMU-8613 in the same terminal clade as *C. candolleanus*. However, the terminal branch length for each taxon deviates from the pattern observed in most geographic variants of the same species in the phylogenetic tree. For example, in *C. asiaticus*, *C. campanulatus*, *C. subsingeri*, *C. singeri*, and *C. subminutisporus*, the two strains representing each species show identical or nearly identical terminal branch lengths (Figure 9). The only geographic variants of the same species that show a difference in terminal branch length similar to that observed in CMU-8613/*C. candolleanus* are those of the species *C. subcacao*. This suggests that strain CMU-8613 is related to *Candolleomyces candolleanus* (syn. *Psathyrella candolleana*), making this the first publicly available, assembled, and annotated genome of this genus.

A comparative analysis of pipelines for basidiomycete genome assembly reveals a clear trend toward hybrid strategies, increasingly favoring the combination of long and short reads to optimize both structural contiguity and base-level accuracy. Nonetheless, current studies indicate that no universally optimal approach exists for basidiomycete genomes, as technical challenges vary with sample type, heterozygosity level, and sequencing depth [13]. For *Clitopilus passeckerianus* and *Phellinotus piptadeniae*, assemblers tailored for noisy long reads, such as Canu and MaSuRCA, have been used, followed by extensive polishing with Pilon using Illumina data [14,15]. These pipelines produced robust assemblies with high BUSCO completeness scores (93.4–93.3%). However, they remained moderately fragmented (N50 < 700 kb), reflecting the limitations of relying on iterative correction procedures without explicit heterozygosity-aware modeling.

Recent assemblies employ advanced strategies to improve structural resolution. For instance, in *Ganoderma boninense*, integrating Hi-C data via the HiRise pipeline enabled a chromosomal-level assembly (N50 > 4 Mb). In contrast, in *Cyathus olla*, a comparable level of contiguity was achieved solely through deep Nanopore sequencing coverage and a robust assembly workflow based on NECAT and Purge_haplotigs [16,17]. These findings demonstrate that long-read sequencing, combined with effective haplotypic collapse algorithms, can compensate for and, in some cases, outperform chromosomal proximity data. The use of HiFi reads in *Armillaria* spp. and *Lentinula edodes* has become the standard for achieving high accuracy in genome reconstruction [13,18]. In particular, in *L. edodes*, a landmark fully phased (haplotype-to-haplotype) assembly was generated using hyphae and single-spore-derived material, enabling the reconstruction of an entire diploid genome. However, this approach demands substantial resources and specialized datasets that are not always accessible.

In this context, the pipeline developed in this study for strain CMU-8613 offers a methodologically balanced and comparatively innovative approach. Unlike previous works that rely on a single assembler or on the direct integration of short and long reads, this approach systematically evaluated the performance of three long-read assemblers, Canu, Flye, and NECAT, and assessed their trade-offs between contiguity and gene completeness. The results align with earlier observations indicating that NECAT generates highly contiguous assemblies (N50 > 1 Mb), whereas Flye achieves superior gene representation (BUSCO > 96%). The observed trade-off between NECAT’s high contiguity and Flye’s superior completeness can be attributed to how each algorithm handles the complex genomic features typical of Basidiomycota. Basidiomycete genomes are often characterized by high levels of heterozygosity and a substantial accumulation of repetitive elements, such as transposons [19]. NECAT uses a global alignment approach that effectively bridges repetitive regions by identifying coverage peaks [20], resulting in longer contigs but potentially collapsing closely related paralogs or alleles, which explains the lower gene completeness. Conversely, Flye constructs a repeat graph designed to preserve alternative paths in complex regions [21]. While this strategy is more sensitive to retaining gene content and resolving structural variants common in heterozygous fungi, it can lead to graph breaks at complex repeats, resulting in a more fragmented assembly but with higher gene recovery. Further analysis with the RepeatScout, LTRharvest, and LTR_STRUCT tools [19] will allow us to determine which of the assembly strategies used here better recovers transposable elements of the here annotated genome.

A distinctive aspect of this study is the implementation of a cross-bridging approach, in which the highly contiguous structural assembly produced by NECAT served as a reference to reorganize the more gene-rich contigs generated by Flye. This internal hybrid approach, not reported in most of the reviewed cases, integrated the strengths of both algorithms, yielding an assembly with an optimal balance between contiguity and completeness. Additionally, the sequential application of long-read-specific polishing tools (Racon, Medaka), followed by short-read polishing with Pilon, effectively corrected residual homopolymer-associated errors, substantially enhancing the overall accuracy of the genome assembly. The rationale for performing four iterations was to reach a convergence point where increases in BUSCO completeness scores and reductions in indel errors plateaued, ensuring maximum consensus accuracy without overcorrection. Subsequently, four rounds of short-read polishing with Pilon were conducted to correct residual homopolymers and frameshifts.

The estimated genome size of *C. candolleanus* ranged from 46.8 Mb (NECAT + Racon) to 59.3 Mb (Canu + scaffolding with the *C. micaceus* genome), depending on the assembly and polishing strategy. In GenBank, the reported genome sizes for *C. eurysporus*, *C. efflorescens*, and *C. aberdarensis* are 70, 33.6, and 60.6 Mb, respectively (Supplementary Table S1). However, these assemblies remain at the scaffold or contig level (500–2300 contigs), lack annotation, and are highly fragmented, with N50 values of only 57–157 kb. These limitations are primarily attributable to the predominant use of Ion Torrent sequencing and medium to low coverage. Therefore, these genomes are not appropriate comparative references for the annotated genome generated in this study and, for the same reasons, were not used as templates during the assembly of strain CMU-8613. The genome of *C. micaceus* was selected as the structural reference because it is the taxonomically closest fully annotated genome available at the chromosome level within the family Psathyrellaceae [22]. Previous phylogenomic studies in Agaricales have demonstrated a high degree of macrosynteny and conservation of gene order among closely related genera within the same family [23,24]. Although *C. micaceus* and *C. candolleanus* are distinct species, their phylogenetic proximity [1] allows the reference to serve as a reliable guide for orienting contigs without imposing artificial adjacencies, as confirmed by the stability of BUSCO scores after scaffolding process.

Beyond the genomes of *Candolleomyces* spp., the family Psathyrellaceae in GenBank includes 14 assemblies unevenly distributed across four genera. The most represented genus is *Coprinopsis*, with five genomes (Supplementary Table S3), followed by *Coprinellus*, with four (Supplementary Table S4), and by *Ephemerocybe* and *Psathyrella*, with two genomes each (Supplementary Tables S5 and S6, respectively). Additionally, a single assembly corresponds to an uncultured Psathyrellaceae taxon (Supplementary Table S6). Most of these genomes are deposited at the scaffold or contig level. Among them, *Coprinellus* and *Coprinopsis*, particularly the model strain *Coprinopsis cinerea*, show the most robust metrics, with N50 values exceeding 3 Mb, fewer contigs, and the use of long-read technologies such as PacBio or Oxford Nanopore. The genome of *C. micaceus* (GCA_951394405.1) is annotated at the chromosome level and was used as a reference in this study to assemble strain CMU-8613. Its size, 52 Mb, is comparable to the upper range of genome estimates obtained for *C. candolleanus*, reaching 59.3 Mb in this study.

At a higher taxonomic level, where more high-quality, annotated genomes are available, genome sizes within the order Agaricales range from 22.12 Mb (*Amanita inopinata*) to 175 Mb (*Tricholoma matsutake*) [25,26]. This broad variation has been linked to lineage-specific expansions and losses of genes associated with ecological transitions, particularly the diversification of substrate utilization strategies among saprotrophic fungi and shifts from saprotrophy to mycorrhizal lifestyles [23,24]. A more detailed functional analysis of the *C. candolleanus* genome will help clarify its saprotrophic potential, a lifestyle to which the species has been frequently attributed. High-quality assemblies and annotations from additional species within the family Psathyrellaceae, especially from *Candolleomyces* and closely related genera, are essential for improving ecological and physiological predictions for this taxonomic group, including assessments of plant biomass degradation capacity and secondary metabolite biosynthesis.

Within Agaricales, mitochondrial genome size varies substantially among species within the same genus and even among isolates of a single species. In commercially important edible members of Agaricales, mitochondrial genome sizes also show considerable variability. For example, species of the genus *Pleurotus* possess relatively uniform mitochondrial genomes ranging from 60,694 to 73,807 bp [27]. In contrast, *Agaricus bisporus* and *Lentinula edodes* have much larger mitochondrial genomes of 135,005 bp and 121,394 bp, respectively. Recent studies have reported mitochondrial genome sizes ranging from 114,236 to 129,263 bp within the family Nidulariaceae [28], as well as an extreme case in *Clavaria fumosa* (family Clavariaceae), which reaches 256,807 bp [29]. The gene content and structure of the mitochondrial genome of *C. candolleanus* strain CMU-8613 are consistent with the smaller mitochondrial genomes found within Agaricales, including those of closely related species such as *C. micaceus* (64,450 bp) [27] and *Coprinopsis cinerea* (42,448 bp; NW003307477.1) [30]. Expansions in mitochondrial genomes within Agaricales have been associated with repetitive regions and introns [27,28], features that are uncommon in these species, including the CMU-8613 strain.

Genome mining has revolutionized natural product discovery, enabling the identification and characterization of biosynthetic gene clusters (BGCs) that encode diverse secondary metabolites [31,32]. Using this approach, He et al. [11] identified a BGC responsible for the biosynthesis of guanacastane-type diterpenes in *P. candolleana*. Through heterologous expression, they demonstrated that the diterpene synthase PsaD catalyzes the cyclization of geranylgeranyl diphosphate, while the monooxygenase PsaA (cytochrome P450) mediates successive oxidation steps leading to guanacastane terpenes. The functional genomic analysis presented here highlights the importance of genome assembly, annotation, and BGC prediction tools for identifying and localizing genes involved in secondary metabolite synthesis in fungi. Depending on the combination of tools used, the number of predicted BGCs in *C. candolleanus* ranges from 15 (NECAT/antiSMASH) to 25 (Fly/FunBGCex), with terpene synthesis (TS) clusters being the most abundant, ranging from 9 to 14 across analyses.

The second major group comprises clusters encoding enzymes involved in the biosynthesis of non-ribosomal peptides and polyketides (NRPS/PKS, including all variants). All prediction tools consistently identified five BGCs. As previously mentioned, no high-quality annotated genome is available for *Candolleomyces/Psathyrella* that would allow a direct comparison with strain CMU-8613. Within the Psathyrellaceae, however, the genome of *Coprinopsis cinerea* harbors nine terpene synthesis (TS) BGCs and 5 NRPS/PKS [33]. Overall, the number of BGCs associated with major metabolite classes in CMU-8613 falls within the range reported for other Agaricales, although their distribution varies among species. For example, in *Laccaria bicolor*, 8 TS and 5 NRPS/PKS are encoded, whereas in *Schizophyllum commune*, 5 and 10 are encoded, respectively. Edible species of great commercial relevance, *Agaricus bisporus* (button mushroom) and *Pleurotus ostreatus* (oyster mushrooms), show the same trend, with 10 and 15 TS BGCs and 8 and 9 NRPS/PKS, respectively [33]. A remarkable exception within Agaricales is the genus *Cyathus*, whose species harbor between 41 and 209 BGCs; among these, 12–95 correspond to terpene biosynthesis and 18–49 to the NRPS/PKS pathway, depending on the species [34]. Increasing the number of annotated genomes from species within the Psathyrellaceae will help determine whether the relatively low number of BGCs observed in CMU-8613 of the family reflects lineage-specific reductions.

One of the biologically relevant findings in this genome is the identification of a biosynthetic gene cluster on Scaffold 6, annotated as a polyprenyl pyrophosphate synthase (PPPS). Detailed analysis of this cluster reveals the co-occurrence of a geranylgeranyl pyrophosphate synthase (GGPPS) and a cytochrome P450 monooxygenase. This genomic organization aligns with the chemical structure of psathyrellanic acid, a known metabolite of *C. candolleanus* with antibacterial properties [8,10]. Theoretically, the GGPPS is required to synthesize the 20-carbon precursor (geranylgeranyl diphosphate). At the same time, the cytochrome P450 monooxygenase would catalyze the subsequent oxidation of the terpene skeleton, a structural feature observed in psathyrellanic acid and guanacastane diterpenes [11]. Therefore, we propose this locus on Scaffold 6 as the putative biosynthetic gene cluster for these bioactive compounds, providing a clear target for future heterologous expression studies.

Species in genera within the order Agaricales have more CAZyme-encoding genes than genera in the orders Russulales, Polyporales, and Boletales [24]. The number of CAZyme-encoding genes in species of *Candolleomyces*, including strain CMU-8613, is consistent with this pattern and exceeds the number of CAZyme genes in *Inonotus obliquus*, a member of the order Hymenochaetales [29]. The number of CAZymes aligns with the saprophytic lifestyle of most *Candolleomyces* species [1], indicating that this taxon has strong potential for use in composting processes and biofuel production [35,36].

The localization of the *MAT-A* and *MAT-B* genes in the genome of strain CMU-8613 indicates that it has a tetrapolar mating system, a condition common in Agaricales and considered ancestral for mating types [37]. However, intraspecific variation in mating systems has been documented [38]; thus, as additional genomes from this genus become available, it will be possible to more precisely determine the mating strategies that generate genetic diversity within *Candolleomyces*.

## 4. Materials and Methods

### 4.1. Study Strain

The strain CMU-8613 was isolated and studied in 2017 in the state of Michoacán from a soil sample collected in a mixed-vegetation area in the municipality of Tarímbaro, Michoacán [39]. The strain is part of the Michoacan University Culture Collection (CMU) of the Microbial Biotechnology and Conservation Laboratory at the Multidisciplinary Center for Biotechnology Studies of the Faculty of Veterinary Medicine and Animal Science at the Universidad Michoacana de San Nicolás de Hidalgo.

### 4.2. DNA Extraction

For maintenance of the study strain and collection of biomass for DNA isolation, solid malt extract agar (CEM) medium (BD Difco™, Sparks, MD, USA) was prepared according to the supplier’s specifications and sterilized at 121 °C and 15 lb/in^2^ for 15 min. Genomic DNA was extracted from actively growing mycelium cultivated on CEM, which was harvested and placed into 2 mL microcentrifuge tubes. DNA extraction was performed using the ZymoBIOMICS™ DNA Miniprep Kit according to the manufacturer’s instructions (https://files.zymoresearch.com/protocols/_d4300t_d4300_d4304_zymobiomics_dna_miniprep_kit.pdf accessed on 5 December 2025).

### 4.3. Illumina Sequencing

Illumina (San Diego, California, USA) sequencing libraries were prepared using the Illumina DNA preparation kit, which includes PCR and tagmentation, along with custom 10 bp dual-unique indices (UDI), targeting an insert size of 320 bp. Sequencing was performed on the NovaSeq 6000 system, with one or more multiplexed runs on shared flow cells, generating paired-end reads of 2 × 151 bp. Demultiplexing, quality control, and adapter trimming were performed with bcl-convert1 (v4.1.5). After sequencing, raw read quality was evaluated using FastQC v0.11.5 (https://github.com/s-andrews/FastQC accessed on 5 December 2025). Adapter sequences and low-quality bases (Phred score < 20) were trimmed with Trimmomatic (v0.40) [40].

### 4.4. Oxford Nanopore Sequencing

Sequencing libraries were prepared from genomic DNA using the Oxford Nanopore Technologies (ONT, Oxford, UK) ligation sequencing kit (SQK-NBD114.24, ONT, Oxford, UK) with the NEBNext^®^ (E7180L, New England Biolabs®, Ipswich, MA, United States) supplemental module, following the manufacturer’s specifications. No additional DNA fragmentation or size selection was performed. Nanopore sequencing was performed on an Oxford Nanopore MinION Mk1B sequencer (ONT, Oxford, UK) with R10.4 flow cells. For high-accuracy base calling (SUP), Guppy1 (v6.4.6) was used for demultiplexing and adapter trimming.

### 4.5. Genome Assembly and Polishing

A hybrid assembly approach was used, consisting of a primary genome assembly with ONT reads using three assemblers: Flye (v2.9.5), Canu (v2.3), and NECAT (v0.0.1) [20,21,41]. The primary assembly was polished with ONT reads using minimap2 (v2.28) [42] and four rounds of Racon + Medaka (V1.5.0/V1.7.2) [43,44]. Short Illumina reads were used for secondary polishing with Pilon (v1.20.1) [45] in four rounds. Duplicate contigs (haplotigs) were removed using purge dups (1.2.6) [46]. The quality and integrity of each assembled genome were evaluated using BUSCO (v5.8.0) (http://busco.ezlab.org/ accessed on 5 December 2025), with the OrthoDB_10 database for Agaricales.

### 4.6. Genome Arrangement

Two genome assemblies were generated using different polishing strategies. The first assembly used the genome of KDTOL0000127, ToLID gfCopMica1 of *Coprinellus micaceus* (GenBank ID: GCA_951394405.1), which also belongs to the order Agaricales and the family Psathyrellaceae and is phylogenetically close to the strain under study. Genomes of *Candolleomyces* available in public databases were excluded due to low assembly quality (Supplementary Table S1). The cross-assembly method was also employed, using the best result from the *C. micaceus* reference to perform a second assembly, aiming to obtain a high-contiguity, genetically complete assembly. Both assemblies were performed using the RagTag tool (v2.1.0) [47].

To assess the structural consistency and gene order of the ordered genome assembly, a whole-genome alignment was performed between *Candolleomyces candolleanus* CMU-8613 and the reference genome of *Coprinellus micaceus* (GCA_951394405.1). The alignment was conducted using Mauve (v2.4.1, snapshot_2015-02-13) [48], employing the Progressive Mauve algorithm with default parameters. Locally Collinear Blocks (LCBs) were identified to visualize conserved homologous regions and evaluate macrosynteny between the two genomes.

### 4.7. Genome Annotation

Structural and functional annotation of the ordered genomes was performed using the Funannotate tool (v1.8.15) [49], adding the Agaricales model (OrthoDB 10) to the initial training set of *C. micaceus* species in Augustus for BUSCO alignment. For functional annotation, the Funannotate database 2023-09-28-003158 was used, employing the annotation files from InterProScan (v5.76-107.0) and Eggnog-mapper (v2.1.13), which were previously obtained from the protein sequences of the structural annotation. The annotated files in GenBank format were subsequently analyzed and compared using the antiSMASH (v6.1.1) and FunBGCEx (Fungal Biosynthetic Gene Cluster Extractor) tools (v1.0.1), both specialized in detecting biosynthetic gene clusters (BGCs) involved in secondary metabolism in fungal genomes. [50,51]. This Whole-Genome Shotgun project has been deposited at DDBJ/ENA/GenBank under the accession JBSUFY000000000. The version described in this paper is version JBSUFY010000000. The raw genome sequencing data and the reported assembly are linked to NCBI BioProject PRJNA1370195 and BioSample SAMN53422976 in GenBank.

### 4.8. Mitochondrial Genome Assembly

The mitochondrial genome assembly was performed using MitoHiFi (v3.2.3) [52], which integrates the MitoFinder module [53] for identifying and annotating mitochondrial sequences. The combined use of both systems enabled the selection of the final mitochondrial contig and ensured the overall quality of the assembly.

### 4.9. Identification of CAZyme-Encoding Genes

Identification of enzymes associated with carbohydrate metabolism (CAZymes) was performed using the dbCAN3 (v4.1.4) platform (HMM-based Automated Carbohydrate-active enzyme annotation) [54] with the protein FASTA files generated during the Funannotate annotation process and those deposited in NCBI for *Candolleomyces* species (Supplementary Table S1). Only high-confidence CAZymes were considered, specifically those annotated by two or more methods, in accordance with the official recommendations of the dbCAN consortium.

### 4.10. Identification of the MAT-A/MAT-B Genes

To identify genes associated with the mating system in the strain CMU-8613 of *Candolleomyces candolleanus*, functional annotations from Funannotate were used, including Pfam and InterProScan predictions incorporated into the GenBank files of the assembled genome. Genes corresponding to the *MAT-A* locus were identified by detecting characteristic domains: the homeodomain (HD1 and HD2), with Pfam PF05920, and the mitochondrial intermediate peptidase (MIP), with InterPro IPR045090. For the *MAT-B* locus, genes associated with GPCR-type pheromone receptors were identified using the following functional identifiers: Pfam PF02076 (fungal pheromone receptor, STE3) and InterPro IPR001499 (G-protein coupled receptor-like). The search was conducted by examining the annotations of each gene in assemblies generated by Flye and NECAT, and by recording the genomic positions and gene orders for each scaffold. The resulting gene organization was graphically represented to evaluate the potential mating system structures.

### 4.11. BLASTn Search and Phylogenetic Analysis

The ITS region (ITS1-5.8S-ITS2) was amplified by PCR, and the resulting sequence was compared with the internal transcribed spacer (ITS) database of fungi and reference materials from the National Center for Biotechnology Information (NCBI, https://www.ncbi.nlm.nih.gov accessed on 5 December 2025) using the BLASTn (Basic Local Alignment Search Tool) program. The sequence helped associate strain CMU-8613 with the genus *Psathyrella* and select a phylogenetically close reference genome for assembly and annotation (see Genome Assembly and Annotation section below). After assembling and annotating the genome, the ITS and large ribosomal subunit (LSU) sequences were identified and selected, then used to perform another BLASTn search as described above, selecting those with the highest identity to the sequences of strain CMU-8613 (Supplementary Table S2 [55,56,57,58,59,60,61,62,63,64,65,66,67,68]). A FASTA file was generated for each study sequence set. Sequences from two species of the genus *Hausknechtia*, a genus closely related to *Candolleomyces* [1], were also retrieved and used as an outgroup. The FASTA sequences for each gene from the BLASTn search were concatenated and aligned using the MAFFT v.7 server [69]. The best evolutionary model for each sequence and the phylogenetic reconstruction via maximum likelihood (ML) were performed on the IQ-TREE web server [70,71]. The robustness of the internal branches in the resulting phylogeny was assessed with 1000 replicates to obtain ultrafast bootstrap (UFBoot) values for each bifurcation. Phylogenetic analysis using Bayesian criteria was conducted with MrBayes (v3.2) [72], running four Markov Chain Monte Carlo (MCMC) chains, starting from a random tree topology and setting a burn-in of 25%. Posterior probability values for each node were then calculated. The final phylogenetic tree was edited using iTOL [73].

### 4.12. Operating Environment

All bioinformatic analyses were performed on a Linux-based computational cluster. Unless otherwise stated, all software tools were run with default parameters. To ensure reproducibility, compute jobs for the most resource-intensive steps—primary assembly, iterative polishing, and quality assessment—were configured with 20 CPU threads and 100 GB of RAM.

## 5. Conclusions

Compared with high-cost pipelines based on Hi-C or PacBio HiFi, the strategy applied to *C. candolleanus* demonstrates that a high-quality genome assembly can be achieved through an appropriate combination of open-source tools and an iterative experimental design. This finding positions the pipeline used in this study within a competitive methodological framework. The comparison of assembly metrics shows that assembly quality depends not only on sequencing technology but also on the pipeline’s ability to integrate three key elements: (i) effective haplotype resolution and heterozygosity control, (ii) a multi-stage hybrid polishing strategy, and (iii) the use of complementary assembly algorithms. In this regard, the pipeline developed for the genome of *C. candolleanus* CMU-8613 serves as a reproducible, cost-effective model for assembling genomes of non-model basidiomycetes, providing a robust foundation for high-resolution genomic, evolutionary, and biosynthetic analyses.

The complete genome of the *C. candolleanus* strain CMU-8613, curated and annotated in this work, enabled robust phylogenetic placement, characterization of its mating system, and elucidation of this species’ biotechnological potential to produce hydrolytic enzymes and secondary metabolites.

## Figures and Tables

**Table 1:** Comparison of the primary assembly based on BUSCO ^1^ results, using the main metrics of the three primary assemblies.

Criterion	Canu	Flye	NECAT
Complete BUSCOs	96.5% (S: 92.5%, D: 4.1%)	96.7% (S: 93.9%, D: 2.8%)	92.4% (S: 90.4%, D: 2.0%)
Fragmented BUSCOs	0.4%	0.3%	0.4%
Missing BUSCOs	3.0%	3.0%	7.2% (worse)
Contig/N50	95 Kb (worst)	183 Kb	291 KB (best)
Number of contigs	1785 (more fragmented)	771	369 (less fragmented)
Total assembly size	58.6 MB (major)	55.9 Mb	47.1 MB (smaller)
Advantages	Good recovery of the genome	Better recovery of complete genes	More continuous assembly
Disadvantages	Highly fragmented assembly	Intermediate continuity	More gene loss, shorter assembly

^1^ The BUSCO values refer to the ‘agaricales OrthoDB_10′ dataset. S: Single copy, D: Duplicates.

**Table 2:** Comparison of polishing strategies.

**NECAT Assembly**								
Polished	C (%)	S (%)	D (%)	F (%)	M (%)	Contigs	Total length (Mb)	N50 Contig
Racon	92.5	90.6	1.8	0.5	7.0	296	46.8	1 Mb
Pilon	92.4	90.4	2.0	0.5	7.1	1352	47.9	91 Kb
Racon + Medaka	92.6	90.6	1.9	0.5	7.0	296	47.3	845 Kb
Racon + Medaka + Pilon	92.6	90.6	1.9	0.5	6.9	296	47.1	842 Kb
**Flye Assembly**								
Racon	96.5	93.9	2.7	0.4	3.1	392	55.6	584 Kb
Pilon	96.6	93.7	2.9	0.3	3.1	1508	56.6	81 Kb
Racon + Medaka	96.7	94.0	2.7	0.4	2.9	386	56.0	623 Kb
Racon + Medaka + Pilon	96.3	93.7	2.6	1.3	2.4	384	55.8	620 Kb

Note: The BUSCO values refer to the ‘Agaricales OrthoDB_10′ dataset set: C: Complete, S: Single-copy, D: Duplicated, F: Fragmented, M: Missing.

**Table 3:** Quality of the genome assembly of strain CMU-8613 after scaffolding with the reference genome of *Coprinellus micaceus* (GCA_951394405.1).

Criterion	Canu (vs. Primary Assembly)	Flye (vs. Primary Assembly)	NECAT (vs. Primary Assembly)
Complete BUSCOs (%)	96.5 (S: 92.4, D: 4.1) (=)	96.7 (S: 94.0, D: 2.8) (=)	92.6 (S: 90.6, D: 2.0) (+0.2)
Fragmented BUSCOs (%)	0.4 (=)	0.3 (=)	0.4 (=)
BUSCOs absents (%)	3.1 (+0.1)	2.9 (−0.1)	7.2 (=)
Contig N50 (Kb)	95 (=)	183 (=)	**291** (=)
Scaffold N50 (Kb)	114 (+19)	251 (+68)	**1000** (+709)
Number of contigs	1785 (=)	774 (+3)	**368** (−1)
Number of scaffolds	1686 (−99)	702 (−69)	**288** (−81)
Total assembled size (Mb)	59.3 (+0.7)	56.4 (+0.5)	47.5 (+0.4)

Note: The values after scaffolding are shown. The symbols in parentheses indicate the numerical difference relative to the primary assembly and denote the direction of change: increase (+), decrease (−), or no change (=) (Table 1). The best N50 values and the lowest number of contigs/scaffolds are in bold.

**Table 4:** Assembly quality after scaffolding with the NECAT assembly as a reference.

Criterion	Canu	Flye
Complete BUSCOs (%)	96.5 (S: 92.3, D: 4.2) (=)	**96.7** (S: 93.9, D: 2.8) (=)
Fragmented BUSCOs (%)	0.4 (=)	0.3 (=)
BUSCOs absents (%)	3.1 (+0.1)	3.0 (=)
Contig N50 (Kb)	95 (=)	183 (=)
Scaffold N50 (Kb)	**2000** (+1905)	**2000** (+1817)
Number of contigs	1782 (−3)	770 (−1)
Number of scaffolds	882 (−903)	**370** (−401)
Total assembled size (Mb)	60.6 (+2.0)	56.4 (+0.5)

Note: The results are compared with the primary assembly (Table 1). The symbols in parentheses indicate the numerical difference relative to the primary assembly and denote the direction of change: increase (+), decrease (−), or no change (=). The best values for complete BUSCO, N50, and the lowest number of scaffolds are highlighted in bold. BUSCO values: C: Complete, S: Single-copy, D: Duplicates.

**Table 5:** Final quality metrics of the polished and sorted assemblies.

Criterion	NECAT Assembly	Flye Assembly	Observation
Complete BUSCOs (%)	92.6 (S: 90.6, D: 1.9)	**96.3** (S: 94.0, D: 2.7)	Flye more complete
Fragmented BUSCOs (%)	0.5	1.3	Similar
BUSCOs absents (%)	6.9	2.4	Flye with less loss
Contig N50 (Kb)	**842**	620	NECAT more continuous (contig)
Scaffold N50 (Kb)	1000	**2000**	Flye more continuous (scaffold)
Number of contigs	**296**	384	NECAT less fragmented (contig)
Number of scaffolds	**286**	365	NECAT less fragmented (scaffold)
Total assembled size (Mb)	47.2	**55.8**	Flye larger

Note: The best values for each metric (except for fragmented and missing BUSCOs, where lower is better) are in bold.

**Table 6:** Comparison of key annotation statistics.

**Metrics**	**Flye** **Assembly**	**NECAT** **Assembly**	**Observations**
Number of Genes	**15, 550**	13, 481	Flye predicts +2069 genes
Number of mRNA	**15, 259**	13, 206	Correlates with more genes in Flye
Number of tRNA	**291**	281	Similar in both
Complete CDS	**15, 088**	13, 063	Mayor gene integrity in Flye
Total exons	**86, 010**	75, 300	Flye has more exons
Multi-section transcripts	**13, 664**	11, 918	Most apparent genetic complexity in Flye
Single exon	1, 595	1, 288	Low proportion in both
Average gene length (bp)	1813.4	1816.4	Very similar in both
Average exon length (bp)	251.9	250.9	Very similar in both
Average protein length (amino acids)	499.1	501.6	Very similar in both

Note: The higher values for each counting metric are bold.

**Table 7:** Comparison of BGC annotation tools.

Parameter	FunBGCex	antiSMASH
BGCs (Flye)	25	16
BGCs (NECAT)	23	15
Main types	Terpenes, NRPS, PPPS, DMATS, UbiA	Terpenes, NRPS, PKS, NRPS-PKS hybrids
Sensitivity	High (detects partial fragments or variants)	Moderate (focused on entire regions)
Structural precision	Minor (possible redundancy)	Mayor (clear definition of boundaries)
Match with known BGCs	High	High

**Table 8:** Comparison of the types of BGCs identified using different assembly tools.

	FunBGCex		antiSMASH	
Type of BGC	Flye	NECAT	Flye	NECAT
Terpene/TC (Class 1, SHC/OSC, AstC)	14	12	10	9
PPPS (Prenyl pyrophosphate synthase)	3	3	–	–
PT (Prenyltransferase type DMATS or UbiA)	3	3	–	–
NRPS-like	2	2	2	2
NRPS	1	1	1	1
NRPS-PKS	1	1	1	1
PKS/NR-PKS	1	1	1	1
Siderophore	–	–	1	1
Total	25	23	16	15

## Data Availability

This Whole-Genome Shotgun project has been deposited at DDBJ/ENA/GenBank under the accession JBSUFY000000000. The version described in this paper is version JBSUFY010000000. The raw genome sequencing data and the reported assembly are linked to NCBI BioProject PRJNA1370195 and BioSample SAMN53422976 in GenBank.

## Supplementary Materials

The following supporting information can be downloaded at: https://www.mdpi.com/article/10.3390/ijms27010509/s1, Table S1: Statistics on the assembly of *Candolleomyces* species genomes deposited in GenBank; Table S2: Data of sequences from *Candolleomyces* and *Hausknechtia* species retrieved from GenBank used in the phylogenetic analysis; Table S3: Statistics on the assembly of *Coprinopsis* species genomes deposited in GenBank; Table S4: Statistics on the assembly of *Coprinellus* species genomes deposited in GenBank; Table S5: Statistics on the assembly of *Ephemerocybe* species genomes deposited in GenBank; Table S6: Statistics on the assembly of *Psathyrella* species genomes deposited in GenBank.

## Abbreviations

The following abbreviations are used in this manuscript:

AA	Auxiliary Activities
CAZymes	Carbohydrate-Active enzymes
CBM	Carbohydrate-Binding Modules
CE	Carbohydrate Esterases
GH	Glycoside Hydrolases
GT	Glycosyl-Transferases
PL	Polysaccharide Lyases
NCBI	National Center for Biotechnology Information
CDS	Protein-coding genes
BGC	Biosynthetic Gene Cluster
COG	Clusters of Orthologous Genes
ITS	Internal transcribed spacer ribosomal region
LSU	Gene for the Large Ribosomal Subunit
