# Supplementary material for: Hybrid Genome Assembly and Annotation of the Basidiomycete Fungus Candolleomyces candolleanus Strain CMU-8613 Using a Cost-Effective Iterative Pipeline
Source: Int J Mol Sci. 2026 Jan 3;27(1):509. doi: 10.3390/ijms27010509 (PMC12786890; doi:10.3390/ijms27010509)
Supplement: Supplementary file 1 [file ijms-27-00509-s001.zip › ijms-4062188-supplementary.pdf]

Supplementary Table S1. Statistics on the assembly of *Candolleomyces* species genomes deposited in GenBank.

	Species		
**Assembly statistics**	*Candolleomyces eurysporus*	*Candolleomyces efflorescens*	*Candolleomyces aberdarensis*
Submitted GenBank assembly	GCA_027579435.1	GCA_027579765.1	GCA_004126415.1
Genome size	70 Mb	33.6 Mb	60.6 Mb
Total, ungapped length	70 Mb	33.6 Mb	60.6 Mb
Number of scaffolds	1,966	565	2,303
Scaffold N50	78.6 kb	157.6 kb	57.4 kb
Scaffold L50	234	611	297
Number of contigs	1,973	611	2,303
Contig N50	78.1 kb	152.4 kb	57.4 kb
Contig L50	235	68	297
GC percent	50	51	50.5
Genome coverage	48x	86x	18.2x
Assembly level	Scaffold	Scaffold	Contigs
Sequencing technology	IonTorrent	Illumina NextSeq	IonTorrent

Supplementary Table S2. Data of sequences from *Candolleomyces* and *Hausknechtia* species retrieved from GenBank used in the phylogenetic analysis.

Species	Voucher	Country	nrITS	nrLSU	Reference
C. asiaticus	LAH36975	Pakistan	OK392606	OQ802843	[55]
C. asiaticus	LAH36809 Type	Pakistan	NR182405	NG229114	[55]
C. brunneopileatus	TBGT18698 Type	India	OQ878348	OR244401	[56]
C. brunneovagabundus	HKAS129659 Type	China	OR711031	OR711047	[57]
C. campanulatus	LAH35719 Type	Pakistan	OQ308881	OQ802837	[58]
C. campanulatus	LAH37657	Pakistan	OQ308882	OQ802838	[58]
C. candolleanus	LAS73030 Neotype	Sweden	KM030175	KM030175	[59]
C. eurysporus	GLMF126263 Type	Vietnam	MT651560	MT651560	[60]
C. luteopallidus	Sharp20863 Type	USA	KC992884	KC992884	[56]
C. niveofloccosus	TBGT18412 Type	India	OQ878345	OR244387	[56]
C. secotioides	AH31746 Type	Mexico	KR003281	KR003282	[61]
C. sindhudeltae	LAH37632 Type	Pakistan	OQ247908	OQ247912	[62]
C. singeri	HMAS258923	China	OR822171	OR822153	[63]
C. singeri	HMAS258924	China	OR822172	OR822154	[63]
C. subcacao	HMJAU37807 Type	China	MW301064	MW301092	[64]
C. subcacao	HMJAU37808	China	MW301065	MW301093	[64]
C. subminutisporus	HMJAU37916	China	MW301067	MW301095	[64]
C. subminutisporus	HMJAU37801 Type	China	MW301066	MW301094	[64]
C. subsingeri	HMJAU37811 Type	China	MG734715	MW301097	[65]
C. subsingeri	HMIAU37913	China	MG734725	MW301098	[65]
C. sulcatotuberculosus	GB:LÖ55-12	Germany	KJ138422	KJ138422	[66]
C. sultanii	LAH35714 Type	Pakistan	OQ308835	OQ801565	[58]
C. tuberculatus	ADK4162	Sweden	KC992886	KC992886	[56]
C. typhae	LÖ21-04	Sweden	DQ389721	DQ389721	[67]
Hausknechtia floriformis	WU22833	Vanuatu	ON745619	ON745615	[68]
Hausknechtia floriformis	WU22832 Type	Vanuatu	ON745613	ON745616	[68]
Hausknechtia leucosticta	HFJAU1526	China	OL435563	OL435566	[68]
Hausknechtia leucosticta	HFJAU1486 Epitype	China	OL435561	OL435565	[68]

Supplementary Table S3. Statistics on the assembly of *Coprinopsis* specie*s* genomes deposited in GenBank.

	**Species**				
**Assembly statistics**	*Coprinopsis cinerea* okayama7#130	*Coprinopsis marcescibilis*	*Coprinopsis strossmayeri*	*Coprinopsis cinerea* AmutBmut pab1-1	*Coprinopsis* sp.
Submitted GenBank assembly	GCA_000182895.1	GCA_004369085.1	GCA_900156845.1	GCA_016772295.1	GCA_020736565.1
Genome size	36.2 Mb	38.9 Mb	33.3 Mb	38.7 Mb	121 Mb
Total ungapped length	36.2 Mb	38.5 Mb	33.3 Mb	38.7 Mb	121 Mb
Number of chromosomes	13	-	-	-	-
Number of organelles	1	-	-	-	-
Number of scaffolds	67	817	622	31	486
Scaffold N50	3.5 Mb	134.3 kb	190.6 kb	2.8 Mb	607.1 kb
Scaffold L50	5	75	45	6	54
Number of contigs	67	1,533	877	31	486
Contig N50	3.5 Mb	87.1 kb	167.4 kb	2.8 Mb	607.1 kb
Contig L50	5	114	50	6	54
GC percent	51.5	49.5	49	51.5	51
Assembly level	Scaffold	Scaffold	Scaffold	Contig	Contig
Sequencing technology	ABI; lab finishing	Illumina	Illumina HiSeq 2500	Oxford Nanopore MinION; Illumina HiSeq	-

Supplementary Table S4. Statistics on the assembly of *Coprinellus* species genomes deposited in GenBank.

	Species			
**Assembly statistics**	*Coprinellus micaceus*	*Coprinellus micaceus* haploid (alternate haplotype of diploid)	*Coprinellus micaceus*	*Coprinellus aureogranulatus*
Submitted GenBank assembly	GCA_951394405.1	GCA_951394415.1	GCA_004369175.1	GCA_027627275.1
Genome size	52 Mb	2.5 Mb	77.4 Mb	58.2 Mb
Total, ungapped length	51.9 Mb	2.5 Mb	77.4 Mb	58.2 Mb
Number of chromosomes	13	-	-	-
Number of organelles	1	-	-	-
Number of scaffolds	98	119	704	4,667
Scaffold N50	4.1 Mb	20.4 kb	315.8 kb	16.7 kb
Scaffold L50	6	42	55	1,121
Number of contigs	168	119	704	4,712
Contig N50	1 Mb	20.4	315.8 kb	16.6 kb
Contig L50	15	42	55	1,129
GC percent	53.5	52.5	53.5	52.5
Genome coverage	428x	428x	74.8x	12x
Assembly level	Chromosome	Contig	Contig	Scaffold
Sequencing technology	PacBio, Arima2	PacBio, Arima2	Illumina	IonTorrent

Supplementary Table S5. Statistics on the assembly of *Ephemerocybe* species genomes deposited in GenBank.

	Species	
**Assembly statistics**	*Ephemerocybe angulata*	*Ephemerocybe angulata*
Submitted GenBank assembly	GCA_013368325.1	GCA_014188975.1
Genome size	59.3 Mb	93.6 Mb
Total ungapped length	59.3 Mb	93.6 Mb
Number of scaffolds	273	517
Scaffold N50	754.4 kb	482.1 kb
Scaffold L50	21	56
Number of contigs	273	517
Contig N50	754.4 kb	482.1 kb
Contig L50	21	56
GC percent	52.5	53
Genome coverage	68.51x	63x
Assembly level	Contig	Contig
Sequencing technology	PacBio RSII	Pacbio

Supplementary Table S6. Statistics on the assembly of *Psathyrella* species genomes deposited in GenBank.

	**Species**		
**Assembly statistics**	*Psathyrella hymenocephala*	*Psathyrella* sp. B2010_1	uncultured Psathyrellaceae
Submitted GenBank assembly	GCA_051997475.1	GCA_051997435.1	GCA_937898395.1
Genome size	57.4 Mb	48.2 Mb	26.2 Mb
Total ungapped length	57.4 Mb	48.2 Mb	26.2 Mb
Number of scaffolds	28	315	7,051
Scaffold N50	4.5 Mb	374.5 kb	4.9 kb
Scaffold L50	6	38	1,667
Number of contigs	28	315	7,051
Contig N50	4.5 Mb	374.5 kb	4.9 kb
Contig L50	6	38	1,667
GC percent	50.5	50.5	53
Genome coverage	100x	100x	8x
Assembly level	Contig	Contig	Contig
Sequencing technology	PacBio Sequel	PacBio Sequel	Illumina HiSeq 2000
